# ESCRT-III function in membrane fission and repair

**DOI:** 10.1038/s41580-025-00909-1

**Published:** 2025-11-26

**Authors:** M. Burigotto, J.G. Carlton

**Affiliations:** 1 Organelle Dynamics Laboratory, The Francis Crick Institute, 1 Midland Road, London, NW1 1AT; 2 School of Cancer & Pharmaceutical Sciences, King’s College London, SE1 1UL

## Abstract

The endosomal sorting complex required for transport (ESCRT) machinery is an evolutionarily conserved multi-subunit protein complex that remodels cellular membranes. Beyond its classical role in endosomal sorting, the ESCRT machinery has been implicated in an ever-growing number of functions, including viral budding, cytokinesis, autophagy, extracellular vesicle release, pruning of synaptic processes, and the repair and closure of holes in cellular membranes. Membrane remodelling functions are typically ascribed to the ESCRT-III subcomplex. In this Review, we discuss recent mechanistic and structural insights into how these proteins assemble and are remodelled to achieve membrane severing. We focus particularly on how ESCRT-III is engaged at different subcellular compartments during both interphase and mitosis to repair and remodel membranes.

## Introduction

**Figure 1:**
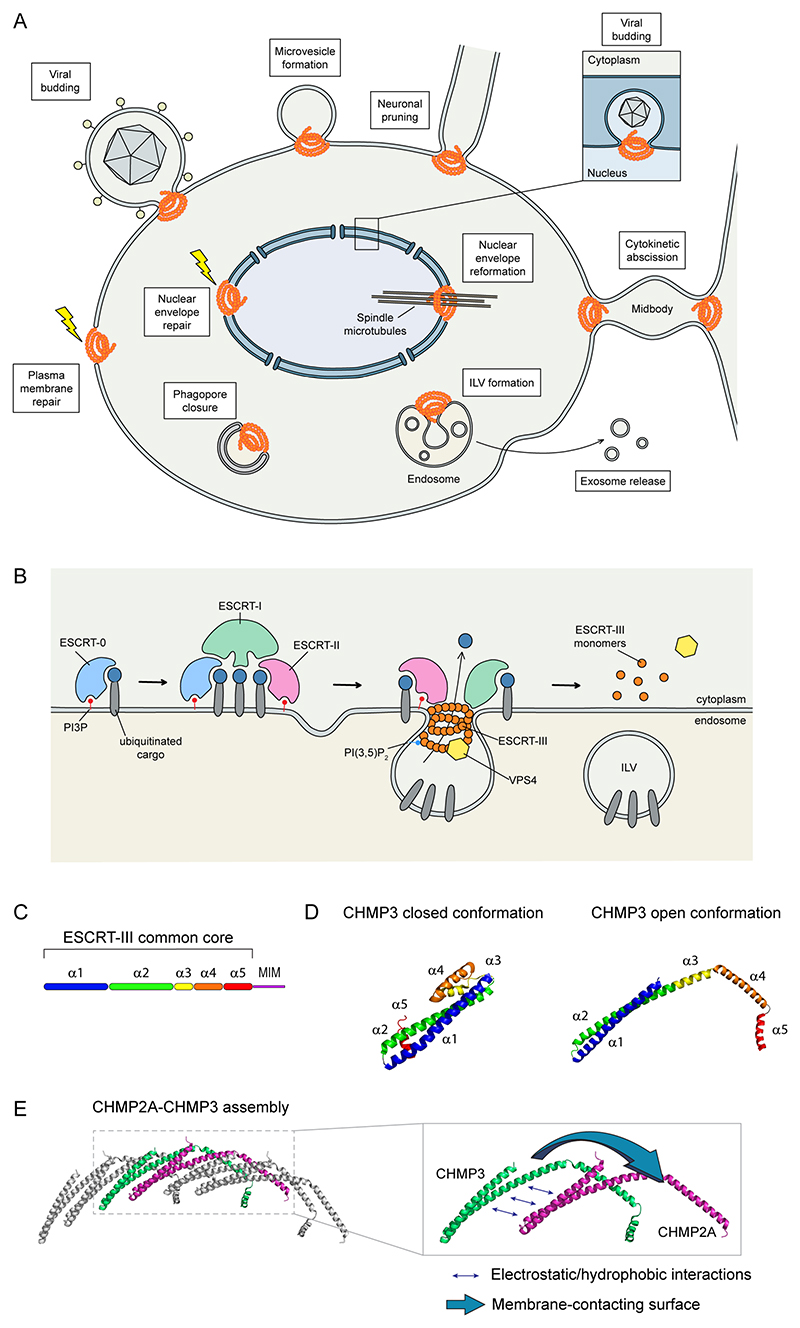
The endosomal sorting complexes required for transport (ESCRT) membrane remodelling machinery. **A**. Following recruitment by adaptor proteins and other ESCRT complexes (see panel b), ESCRT-III-dependent membrane remodelling drives a variety of processes including viral budding, cytokinesis, nuclear envelope regeneration, membrane repair and intra-endosomal vesicle (IEV) formation. **B**. One classical ESCRT function is the formation of IEVs. Here, ESCRT complexes are recruited to the endosome by binding phosphatidylinositol-3-phosphate (PI3P). ESCRT-0 initiates the selection of ubiquitinated cargo. ESCRT-I and-II have additional ubiquitin-binding motifs and interact with these cargoes, whereas ESCRT-0 and ESCRT-III recruit de-ubiquitylating enzymes, which remove ubiquitin before the formation of the IEV. ESCRT-0, ESCRT-I and ESCRT-II (along with accessory factors) are thought to recognise and concentrate ubiquitylated receptors. The subsequent recruitment of ESCRT-III and vacuolar protein sorting 4 (VPS4) to the endosomal surface (along with contributions from the ESCRT-I and probably ESCRT-II complexes)^100^ drives membrane deformation and fission, eventually generating IEVs. ESCRT-III is a multisubunit complex comprised of protein monomers of the family of charged multivesicular body proteins (CHMPs), and a related ESCRT-III protein called increased sodium tolerance-1 (IST1) that polymerise to form membrane-remodelling filaments. Please see Supplementary Table 1 for a full listing of ESCRT components in select organisms. The activity of VPS4 disassembles ESCRT-III filaments and recycles ESCRT monomers to the cytoplasm. Throughout this process, conversion of PI(3)P to phosphatidylinositol-3,5-bisphosphate (PI(3,5)P_2_) may facilitate ESCRT activity. **C**. Cartoon depicting the secondary structure of CHMP3 and highlighting the 5-helix ESCRT core structure and the C-terminal MIT-interacting motif (MIM) domain, responsible for the interaction with VPS4. **D**. Ribbon diagram of CHMP3 in its closed (inactive) (Protein Data Bank entry (PDB ID): 3FRT) and open (that is polymerization-competent) conformation (PDB ID: 7ZCG). **E**. Cartoon depicting part of an ESCRT-III polymer composed of three copies of a CHMP2A–CHMP3 heterodimer (PDB ID: 7ZCG). The close-up shows a single CHMP2A–CHMP3 heterodimer, in which the two proteins have been spatially separated to enhance clarity. The closed-to-open conformational change enables ESCRT-III monomers to make contacts with neighbouring monomers through a combination of electrostatic and hydrophobic interactions between adjacent *α*1–*α*2 cores, driving the formation of a filament. Membrane interaction is driven by residues at the tip of *α*1 and the elbow formed in the open conformation between *α*3 and *α*4. These sites of membrane interaction are observed in the CHMP2A–CHMP3 hetrodimer described above^24,25^, in CHMP1B^20^ and in the yeast CHMP4 orthologue, Snf7 (refs. ^23,34^), although additional membrane interaction surfaces in *α*2 of Snf7 have also been described^34^.

The ability of a cell to remodel its membranes is an essential function that enables both normal growth, homeostasis, as well as response to cellular damage. One of the main membrane remodelling systems in eukaryotes is the endosomal sorting complex required for transport (ESCRT) machinery. This highly conserved protein machinery was first discovered in yeast as a crucial regulator of cargo sorting on endosomes^1–3^. It is now well established that the ESCRT machinery is involved in a wider range of cellular processes, such as budding of enveloped retroviruses, cell division, exosome and microvesicle biogenesis, autophagy, and membrane repair, to name a few (reviewed in^4^) (Figure 1A). Common to all these functions is a series of membrane-shaping events that involve the dynamic assembly of ESCRT-III proteins into filaments of different morphologies, their localisation to membrane neck structures and their reorganisation to constrict and sever these membrane necks, thereby separating membranes that were originally connected.

Canonically, the ESCRT machinery consists of 3 complexes, ESCRT-I, -II, -III, which act in concert with the AAA ATPase vacuolar protein sorting 4 (VPS4) and a variety of auxiliary proteins. Whereas not all ESCRT-mediated processes use the full complement of ESCRT complexes, degradative cargo sorting on endosomes is a well characterised example involving the sequential engagement of all of them, where each of the complexes has a distinct role (Figure 1B). This process begins with capture of ubiquitinated transmembrane domain-containing cargo on endosomes by a complex of hepatocyte growth factor-regulated tyrosine kinase substrate (HRS) and signal transducing adapter molecule-1 (STAM-1) and STAM-2. Whilst not originally identified as an ESCRT complex, the HRS–STAM complex has become known as ESCRT-0, as it acts as an endosomal adaptor for the ESCRT machinery allowing degradation of cargo through the formation of multivesicular bodies (MVBs)^5–7^. In yeast, ESCRT-0 is comprised of Vps27 and Hse1. Throughout this Review, we will primarily use nomenclature from human ESCRT proteins with names from other organisms used to illustrate specific findings (Supplementary Table 1).

Downstream of ESCRT-0, ESCRT-I (composed of tumor susceptibility gene 101 protein (TSG101), VPS28, VPS37, and either multivesicular body sorting factor 12 (MVB12) or ubiquitin-associated protein 1 (UBAP1)) and ESCRT-II (formed by ELL-associated protein of 30 kDa EAP30 (also known as SNF8), EAP20 (also known as VPS25), and EAP45 (also known as VPS36) machineries are sequentially recruited. Together, these macromolecular complexes facilitate the transfer of ubiquitinated cargos to intra-endosomal vesicles, which are eventually generated by ESCRT-III (Figure 1B). ESCRT-associated deubiquitinating enzymes including associated molecule with the SH3-domain of STAM (AMSH, also known as STAMBP) and ubiquitin carboxyl-terminal hydrolase 8 (USP8) or Doa4 in yeast, act to recycle ubiquitin from cargo prior to intra-endosomal vesicle (IEV) formation^8–11^.

The engagement of ESCRT-III is the convergence point for all ESCRT-mediated processes, many of which can proceed without the involvement of the upstream assemblies, and it is this machinery that is thought to provide the mechanical force that drives membrane remodelling. The functional importance of ESCRT-III is underscored by its conservation across the tree of life, with ESCRT-III-like proteins identified in archaea and bacteria^12–14^, suggesting an evolutionary origin that predates the last universal common ancestor (LUCA). In mammals, there are 8 different ESCRT-III proteins of the charged multivesicular body protein (CHMP) family, of which some are present in different isoforms (CHMP1A, CHMP1B, CHMP2A, CHMP2B, CHMP3, CHMP4A-C, CHMP5, CHMP6, CHMP7), and increased sodium tolerance protein 1 (IST1) (Supplementary Table 1). Differently from the other ESCRT complexes, ESCRT-III is only transiently assembled^15,16^. Its ability to operate in different cellular contexts stems from the remarkable conformational plasticity of its subunits, which can adopt a diverse array of filamentous structures. This structural flexibility, combined with the intrinsically dynamic processes that ESCRT-III proteins carry out, has made it challenging to arrive at a thorough description of how these polymers are assembled and remodelled to achieve membrane severing.

In this Review, we will provide a comprehensive overview of the current understanding of how ESCRT-III proteins assemble and remodel membranes, highlighting the most recent findings made possible by the latest advancements in structural and biophysical techniques. In addition, as the maintenance of membrane homeostasis is key for the correct functioning of cells and its deregulation is associated with pathological conditions (Box 1), we will discuss the latest insights into how ESCRT-III proteins act mechanistically at different subcellular compartments to repair membrane damage.

### Structural and mechanistic insights into ESCRT activity

All known ESCRT functions require the activity of ESCRT-III polymers to perform membrane deformation and scission. Individual ESCRT-III proteins are inactive monomers, which, once activated, form oligomeric polymers. These polymeric filaments have an intrinsic curvature and an ability to bind to membranes, allowing changes in filament geometry to be imposed on these underlying membranes. New insights from structural and in vitro reconstitution studies are starting to reveal how the flexible nature of ESCRT-III proteins enables variable filament geometries that can enable the shaping and fission of cellular membranes.

#### ESCRT-III structure

Structural analyses of ESCRT-III proteins^17–21^ have shown that they share a common core architecture consisting of five *α*-helices, although some of these are not unambiguously defined in certain members of the CHMP family (Figure 1C). Additionally, some ESCRT-III proteins contain additional short N-terminal helices which are involved in membrane binding and may help this filament remodel membranes^22–25^. In contrast to the other ESCRT-III members, CHMP7 has an extended N-terminus containing tandem winged helix domains and shows high homology with the ESCRT-II subunit VPS25, whereas its C-terminal part has a classical ESCRT-III-fold^26,27^. These data suggest that CHMP7 is a chimeric ESCRT-II–ESCRT-III protein. IST1 contains an N-terminal ESCRT-III-like domain and an extended C-terminus with distal VPS4-interaction motifs^19,28^, suggesting that it may recruit additional proteins to an ESCRT-III polymer or allow ESCRT-III to operate in other cellular processes such as endosomal recycling^29,30^.

The determination of two different structures for CHMP3 (refs. ^17,19,25,31^) indicated that ESCRT-III monomers can adopt two distinct conformations. In the closed conformation, helix *α*5 folds back onto a four-helix bundle composed of an *α*1–*α*2 helix hairpin aligned against helices *α*3 and *α*4^19^. It is thought that to prevent unscheduled polymerization, soluble ESCRT-III monomers adopt this closed conformation, in which intramolecular contacts lock them into an autoinhibited state (Figure 1D, left). Truncation studies supported the idea that C-terminal sequences are critical to this, as expression of truncated CHMP proteins results in accumulation of ubiquitinated cargos on enlarged endosomes and inhibits viral budding^17,32,33^. The transition between closed and open state involves a conformational rearrangement that generates a continuous helix formed by helices *α*2 and *α*3, giving rise to a highly elongated structure^20,34^ (Figure 1D, right). As described for vacuolar-sorting protein Snf7 (the yeast paralogue of CHMP4), this intramolecular conformational change exposes both a cationic membrane-binding surface and a hydrophobic and electrostatic surface thought to be involved in protein–protein interactions that enable individual monomers to nest against each other to build a filament^20,25,34^ (Figure 1E). The finding that polymeric Snf7, and repeating units of CHMP2A–CHMP3 in the polymeric structure are found in their open conformation supports this model. Moreover, cross-linking mass spectrometry data of CHMP7 showed that the monomeric protein is found in a closed configuration and that when it is polymerized there is no interaction between its N- and C- terminal domains, supporting a closed–inactive versus open–polymerized model for this protein^35^.

Physiologically, the conversion between these conformations is promoted by protein–protein interactions between ESCRT-III subunits and binding partners that are resident to the target membranes, which act as site-specific nucleation factors^36,37^. For instance, on the limiting membrane of MVBs, binding of ESCRT-II to the myristolated ESCRT-III protein Vps20 transitions it from the closed state to the open one, thereby kickstarting ESCRT-III polymerization by engaging Snf7 (ref. ^36,38–41^). A parallel pathway involving BroI binding to Snf7 directly can similarly activate ESCRT-III polymerisation^36^.

#### Assembly and polymerisation on membranes

Upon extension of their helix hairpin, ESCRT-III monomers are able to polymerize, giving rise to metastable oligomeric filaments on membranes. Most ESCRT-III-driven processes involve the resolution of membrane necks of negative membrane curvature, i.e., those that project out of the cytoplasm (Fig. 1a). In some cases, a CHMP1–IST1-containing ESCRT-III complex can assemble on positively-curved membranes^20^, suggesting that complexes containing these subunits control stabilisation or resolution of membranes projecting into the cytoplasm. ESCRT-III can also assemble on flat membranes^42^, although whether these represent functional structures or are intermediates as part of a three-dimensional transition is unclear. Once formed, ESCRT-III polymers are progressively remodelled through the activity of the VPS4 AAA ATPase^16,43,44^. As ESCRT-III acts in different subcellular compartments and contexts, it is not surprising that a variety of stoichiometric ESCRT-III assemblies have been described so far. This could not only be a way to adapt to different surfaces but also to provide opportunities for specific functions and regulation.

ESCRT-III has been shown to polymerise into homo- or hetero-oligomers, which assemble into single- or multi-stranded filaments, adopting many different structures that range from flat spirals to tubes. Both spirals and tubes were first discovered by imaging the plasma membrane of cells overexpressing CHMP4A or CHMP4B^42^ and similar structures were also described upon overexpression of full-length CHMP2B^22^. Furthermore, many ESCRT members have been found to polymerise in vitro, even in the absence of membrane support^19,45–51^. Interestingly, helical structures, whose presence depends on CHMP2A, were revealed by cryogenic electron tomography (cryo-ET) at the midbody of dividing cells^52^, and 3D stochastic optical reconstruction microscopy (STORM) demonstrated that endogenous IST1 spirals were present in the intercellular bridge during cytokinesis^53^, indicating that these polymers are not a mere consequence of protein overexpression but can be formed at physiological levels and contexts. In support of a direct role of these structures in shaping membranes, in vitro co-incubation of different ESCRT-III proteins (CHMP2A and CHMP3, or CHMP1B and IST1) together with supporting bilayers gives rise to co-polymers, which could deform membranes into evaginated tubular or cone-shaped structures^20,25,46,49^ reflecting that seen in cells ^42,54^.

ESCRT-III polymers have extensive positively-charged surfaces^20,25,34^ that are thought to stabilize curved membranes. This is in line with the observation that negatively-charged membranes are required for ESCRT-III assembly and that the mutation of basic amino acids in a dominant-negative CHMP3 completely reversed its dominant-negative behaviour, probably preventing its binding to the membrane^17^. Although ESCRT-III was thought to assemble preferentially on negatively-curved membranes, it is now clear that these proteins can polymerise also on membranes with positive or flat geometries suggesting that the type and degree of membrane curvature can dictate the pattern of ESCRT-III assembly ^20,21,34,55,56^. Snf7 and CHMP4 monomers polymerize into flat spirals ^48,50,51^. Although these filaments can undergo deformation during their growth and accumulate elastic energy^51^, in vitro experiments have shown that the presence of a single spiralling filament is unlikely to drive membrane deformation on its own^48^. Addition of Vps24 and Vps2 subunits to Snf7 spirals not only leads to the formation of an additional Vps24–Vps2 co-polymer alongside the Snf7 sprial^16^ but also gives rise to a minimal system capable of deforming a membrane from a flat surface into a hollow helical tube^48^.

Thanks to cryo-ET and subtomogram averaging, it has been shown that the Snf7–Vps24–Vps2 and CHMP4–CHMP3–CHMP2 co-polymeric helical tubes interact with membranes through multiple interfaces and are likely under elastic stress^57,58^. The extreme N-terminus of many ESCRT-III subunits is thought to anchor the filament in membranes, and opening the *α*3–*α*4 elbow presents a series of basic residues for membrane interaction (Figure 1E). These data show that the ESCRT filament is in close apposition to the membrane, which may exclude transmembrane proteins, allowing the filament to operate as a diffusion barrier^59^. The presence of loose electrostatic interactions between filaments in a CHMP2A–CHMP3 polymer^25^ (Figure 1E) and in Snf7 spirals^34^ are thought to facilitate filament sliding relative to each other. This might allow these filaments to undergo dynamic changes in the way they interact with membranes, to accommodate alternate interaction modes and different geometries as the polymer is remodelled. In support of this, filament tilt and twist has been observed for the ESCRT-III-like cyanobacterial vesicle-inducing protein in plastids 1 (Vipp1) filaments, enabling their transition from planar to 3D-architecture^60–62^, and coarse-grained simulations indicate that alterations in the geometry of the membrane-binding surface of an ESCRT-III filament can drive shape changes and fission of the underlying membrane^63^.

Recent data using high-speed atomic force microscopy have challenged the classical notion that CHMP4 assembles only on flat surfaces, suggesting that Snf7 spirals can adapt to different membrane geometries and when grown on supported lipid bilayers on non-rigid polydimethylsiloxane supports, they can also undergo buckling, suggesting that Snf7 spirals may be sufficient to induce membrane deformation even in the absence of other ESCRT-III proteins^64^. These apparent discrepancies in how ESCRT-III polymers assemble and interact with the underlying membrane raise the exciting hypothesis that a versatile ESCRT machinery could accommodate different ways of performing similar actions depending on the cellular context, such as the conformation, lipid composition, rigidity or tension of the membrane to be severed. Finally, high-resolution electron tomographic imaging has also demonstrated that the membrane underneath polymerised ESCRT-III filaments was thinned^21,25^ and lipids in these bilayers were reorganised^65^, pointing to the fascinating possibility that the very act of ESCRT-III polymerisation alters the biophysical properties of the underlying membrane. Understanding how membrane lipids are reorganised during the fission process, both in cells and with high-resolution structural biology approaches, will be an important next step for understanding how ESCRT-III separates membranes.

#### VPS4 activity and filament disassembly

Differently from cytoskeletal proteins such as actin and tubulin, ESCRT-III does not derive energy by directly hydrolysing nucleotides. Instead, ESCRT-III filaments are actively remodelled by the VPS4 enzyme, which catalyses the disassembly and recycling of their monomers^45,46^. Yeast have a single *VPS4* gene, whereas mammals have 2 paralogues (*VPS4A* and *VPS4B*)^66^. Although some VPS4A-^67,68^ and VPS4B-specific^69^ functions have been reported, the two isoforms seem largely redundant in terms of membrane remodelling activity, with VPS4A showing a higher affinity than VPS4B for ESCRT-III proteins^70^.

VPS4 is a AAA ATPase whose active form is a hexamer^71^. By using the energy derived from ATP hydrolysis, VPS4 translocates ESCRT-III proteins through its central pore^72,73^, causing unfolding of these substrates^74^. VPS4 activity in recycling ESCRT-III monomers is dependent on the presence of a microtubule-interacting and transport (MIT) domain at its N-terminus. The MIT domain directly binds conserved short peptide sequences called MIT-interacting motifs (MIMs) found at the C-terminus of most ESCRT-III components^75–78^ (Fig. 1c). MIT and MIM domains have been reported to interact in many distinct ways: For example, CHMP1A’s MIM resides in a helix, which contacts VPS4A’s MIT in the groove between its helices 2 and 3, whereas CHMP6’s MIM binds VPS4A’s MIT in an extended conformation in the groove between its helices 1 and 3 (ref.^70^).

In addition, VPS4 directly interacts with an ESCRT-III-associated protein called vacuolar protein sorting-associated protein 1 (VTA1, also known as LIP5) and this potentiates both VPS4-ATPase activity and hexamer assembly^79–81^. VTA1 presents a pair of tandem MIT domains involved in ESCRT-III binding^82^ and as VPS4 is able to bind only some ESCRT-III subunits (i.e., CHMP1, CHMP2, CHMP6, and IST1) with relatively high affinity, it is thought that VTA1 supports VPS4 activity by providing additional MIT sites for proteins that bind the ATPase less tightly (i.e., CHMP4 and CHMP3)^70^. In this way, VPS4–VTA1 complexes can make use of many MIT domains, not only increasing avidity for ESCRT-III proteins but also rendering VPS4 able to remodel ESCRT polymers with many different compositions.

#### Remodelling of ESCRT-III filaments

**Figure 2:**
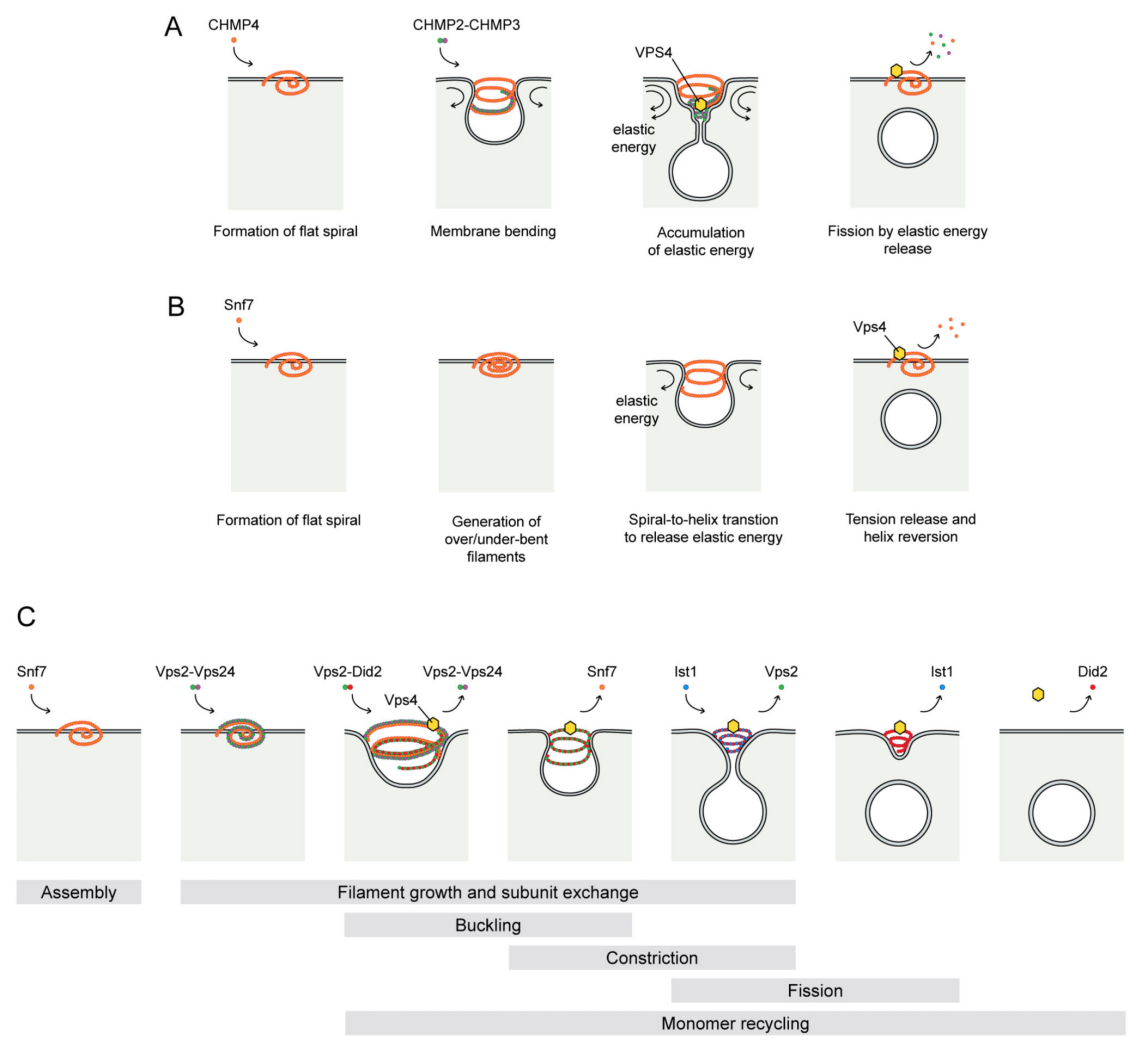
Models of ESCRT-III-mediated membrane fission. **A**. According to the dome model, a spiral filament of charged multivesicular body protein 4 (CHMP4) is capped by a co-polymer of CHMP2–CHMP3 to create a conical surface that narrows membranes to a point of fission. **B**. The buckling model proposes that elastic energy stored by a polymerising filament of Snf7 (yeast CHMP4) is released through an out-of-plane transition (arrows) that leads to membrane deformation. Destabilisation of filaments in the neck and/or reversion of the buckling may provide the energy for fission. **C**. In the subunit exchange model, sequential incorporation and removal of ESCRT-III subunits in a defined order mediate membrane deformation, narrowing of the neck and fission. The ESCRT-III-associated ATPase VPS4 facilitates subunit exchange to enable this remodelling. **D**. The different activities proposed by the previous models can be integrated to generate a hypothetical description of ESCRT-III functioning, in which assembly of a flat filament that could be remodelled to a 3-dimensional helix through dynamic subunit exchange leads to membrane reshaping, the generation of elastic energy and the narrowing of the membrane neck to a point from which it becomes energetically favourable to undergo fission. These predictions are reflected by coarse-grained simulations that show that changes in geometry of the modelled ESCRT-III polymer can induce membrane deformation and that these filament geometry transitions can drive model IEVs to fission^63^.

The ability of ESCRT-III proteins to form polymeric filaments of varying sizes and geometries is instrumental for membrane remodelling as, once assembled, these filaments show an intrinsic tendency to curve the underlying membrane. Nonetheless, whilst coarse-grained simulations demonstrate that directed alterations to the geometry of a polymerising membrane bound ESCRT-III filament can drive membrane deformation and fission^63^, the diverse array of topologies identified has sparked debate about the exact process through which membrane scission occurs. Several non-mutually exclusive models have been proposed based largely on observations made in vitro and are discussed more fully elsewhere^83,84^. In the following we briefly discuss the three individual models shown in Fig. 2.

The dome model^85^ : This model is based on the core ESCRT-III subunits, CHMP4, CHMP3 and CHMP2. According to this model, CHMP4 recruitment leads to the formation of a flat spiral which, while growing, squeezes the supporting membrane, generating an initial membrane bud. CHMP2 and CHMP3 are subsequently recruited and their polymerization at this forming neck leads to the generation of a tubule, whose diameter is progressively reduced by the activity of VPS4^43,46^. The growth of this hemi-spherical cap structure leads to an increase in membrane bending and progressive accumulation of elastic energy which, eventually, lead to stress relaxation by fission of the membrane neck (Figure 2A).

The buckling model^51,86^ : This model stems from the observation that Snf7 and Vps32, form flat curved filaments on membranes^50,51,87^ and the assumption that these polymeric filaments present a preferred curvature. According to this model, continuous polymerization of this filament gives rise to densely-packed spirals in which the inner part of the spiral is over-curved, whereas the growing outer part is under-curved. As seen in a spiral spring, the accumulation of this elastic energy leads to an out-of-plane deformation which transforms the flat spiral into a helix, allowing the filament to adopt its preferred curvature, thus giving rise to membrane invaginations through a buckling mechanism^51^. It is possible that disassembly of the filaments lining the membrane neck could destabilise this neck, releasing the accumulated tension and inducing membrane fission^88^ (Figure 2B).

Subunit exchange model^16,89^ : This model relies on the ability of VPS4 to exchange monomers in existent filaments. Here, VPS4-dependent exchange of ESCRT-III subunits, incorporating monomers with different properties, would induce filament constriction, changes in their curvature, generation of helical tubes and, eventually, narrowing of the membrane neck to a fission point (Figure 2C). Tests of this model have been performed via sequential subunit addition in-vitro with patch spreading experiments on supported bilayers showing that ESCRT-III activity was kickstarted by the polymerization of Snf7, which gave rise to a planar single-filament spiral. This filament was rigidified by the oligomerization of a Vps2–Vps24 heterofilament. On liposomes, incorporation of a separate heterofilament comprising Vps2 and the Did2, the yeast orthologue of CHMP1, alongside Vps4-dependent removal of the existing Vps2–Vps24 filament promoted an inward membrane bending effected by a tilt in the geometry of the Vps2–Did2 filament. Subsequent disassembly of the Snf7 filament and replacement of the Vps2–Did2 filament by a Did2–Ist1 co-polymer led to further constriction of invaginated membrane tubules on liposomes and resulted in membrane fission. This entire process was dependent on the activity of Vps4, which drove unidirectional progression through this series of regulated subunit exchanges^89^. These in vitro data are supported by the fact that Vps4-dependent ATP hydrolysis can narrow polymers of CHMP2A-CHMP3 (refs. ^25,43,46^) and can apply force to, and sever, membrane nanotubes from the inside, in the presence of the core ESCRT-III subunits Snf7, Vps24 and Vps2 (ref. ^90^).

Whilst the subunit exchange model reconciles much evidence obtained through structural analysis, in vitro reconstitution and molecular modelling, it is still unclear whether this series of events is shared by ESCRT-III driven membrane remodelling in vivo. Unlike the buckling or dome models, the subunit-exchange model provides a role for Vps4 throughout the filament remodelling process, which is consistent with its recruitment dynamics in cells^15,16^. However, lattice light sheet imaging in *S. cerevisiae* suggests coordinated, rather than sequential, recruitment of Snf7, Vps24 and Vps2 during endosomal sorting^15^ and whilst ordered recruitment of ESCRT-III components was observed during spindle pole body extrusion in *S. pombe*, Ist1 was recruited alongside Snf7, rather than at the end of a recruitment cascade^91^. Additionally, Did2 and Ist1 deletion present only minor cargo-sorting phenotypes in yeast^92,93^, and IST1 or CHMP1 depletion does not affect all ESCRT functions in mammalian cells, suggesting that they may be dispensable for core ESCRT-III activity ^28,94^. Indeed, many studies have demonstrated that a minimal module composed of CHMP4–CHMP2–CHMP3 (Snf7–Vps2–Vps24 in yeast) and VPS4 seems sufficient to perform membrane fission^25,95^.

#### Membrane fission

These models share similarities in the accumulation of elastic energy in the narrowed and highly curved neck of the forming vesicle which may be released through fission to achieve membrane separation^96,97^. Indeed, during HIV-1 release, CHMP4 and VPS4 disassemble before membrane fission occurs^98^, suggesting that the role of ESCRT-III may be to scaffold a membrane neck that is narrow enough for biophysical principles to drive the fission process to completion. Additional mechanisms for protein-scaffolded membrane fission involve the insertion of hydrophobic wedges into the bilayer’s outer leaflet^97^. Whilst this often produces positive curvature, it is possible that insertion of the amphipathic N-terminal helices found in Snf7, Vps2 and Vps24 (ref. ^23^) and their mammalian equivalents^22,25^ helps destabilises membranes to aid fission. Lastly, although addition of IST1 to a positively-curved assembly of CHMP1B on lipid tubules could constrict this membrane^20,21^, these constrictions did not progress to fission and it is likely that an additional event, such as the presence of a frictional force, is needed to complete this process^99^.

Finally, these models for ESCRT-III-dependent filament remodelling are not incongruent; it is possible that elements of all are correct and one can imagine how the entire process of deformation and fission is achieved through initial flat growth of an ESCRT-III filament, dynamic remodelling of filament architecture, tilt and composition driven by VPS4-dependent subunit exchange to allow a transition that deforms the membrane and allows progression to a dome-like structure inside the neck to narrow and destabilise membranes sufficiently for progress to fission (Figure 2D) (see below). These predictions are reflected by coarse-grained simulations that show that changes in geometry of a modelled ESCRT-III polymer can induce membrane deformation and that these filament geometry transitions can drive model IEVs to fission ^63^.

#### Deformation versus fission

A final aspect that should be considered is the fact that not all ESCRT-remodelled membranes are equivalent, both in terms of composition and topology. The question of how an ESCRT-III machinery can deform a flat membrane away from the cytosol is fascinating. However, the majority of ESCRT-III-dependent membrane fission events involve the scission of pre-formed membrane necks, for example, those formed by the budding of viral Gag proteins, or when double-membraned sheets of nuclear envelope or autophagosomes meet, or those that are generated by the actions of the cytokinetic apparatus that pull a midbody into a thin membrane tube for abscission. IEV biogenesis is somewhat atypical in the panoply of ESCRT-III driven events in that an initial deformation of a flat membrane to form this neck is required. In line with this observation, not only ESCRT-III-intrinsic abilities, but also scaffolding functions ascribed to ESCRT-I^100^ or the role of biophysical condensates^101^ may contribute to the shaping of flat endosomal membranes into necks for ESCRT-III to sever. It will be important for future research to separate activities driving membrane shaping from those driving membrane fission.

### ESCRT-III function in membrane repair

Eukaryotes rely on membrane compartmentalization not only to control interactions with the extracellular environment but also to conduct specialized internal processes. As maintaining the integrity of these membranes is essential for cellular function, cells have evolved a series of repair mechanisms, which are activated by external insults and in different physiological contexts. Amongst these, ESCRT-III has a central role in repairing a wide range of cellular membranes. Current models posit a ‘sense, plug and seal’ mechanism in which damage is detected, the site is stabilised and membrane continuity is restored^102^. When discontinuities occur, ESCRT-III assembles at these damaged regions where it mediates membrane sealing and helps preserve cellular and organellar compartmentalisation. In the following sections we describe different conditions under which ESCRT-III-mediated membrane repair is required.

#### Nuclear envelope repair

In eukaryotes, the genomic DNA is separated from the cytoplasm by the nuclear envelope, a double phospholipid bilayer membrane. The nuclear envelope functions not only as a barrier but also as a filter, regulating the movement of proteins and RNA molecules between nucleoplasm and cytoplasm. This filter function is provided by nuclear pore complexes (NPCs), multimeric protein channels that enable the flow of molecules in and out of the nucleus. The nuclear envelope is divided into an outer nuclear membrane (ONM), which is in continuity with the ER, and an inner nuclear membrane (INM). The INM contains transmembrane proteins, which associate with the nuclear lamina, a mesh of intermediate filaments, which provide structural support to the nucleus, maintaining its shape and plasticity.

The nuclear envelope can undergo rupture both in a physiological, regulated manner (i.e., during an open mitosis) and because of damage derived from mechanical forces (such as during constrained migration)^103–106^, DNA damage^107^ or envelope weakening due to loss of nuclear envelope components (e.g., as seen in laminopathies)^108,109^. In both scenarios, its integrity must be re-established to restore nucleocytoplasmic compartmentalisation. The ESCRT-III complex, most notably the ESCRT-II–ESCRT-III hybrid protein, CHMP7, has been implicated in the sealing of discontinuities in the nuclear envelope. The absence of ESCRT-III activity during late anaphase results in persistent fenestrations in the reforming nuclear envelope, compromising its barrier function and leading to DNA damage^110,111^. Similarly, during interphase nuclear envelope ruptures, CHMP7 (ref. ^112^) and other ESCRT-III members^105,106^ are recruited to sites of damage and needed for repair.

#### Nuclear envelope regeneration during cell division

**Figure 3:**
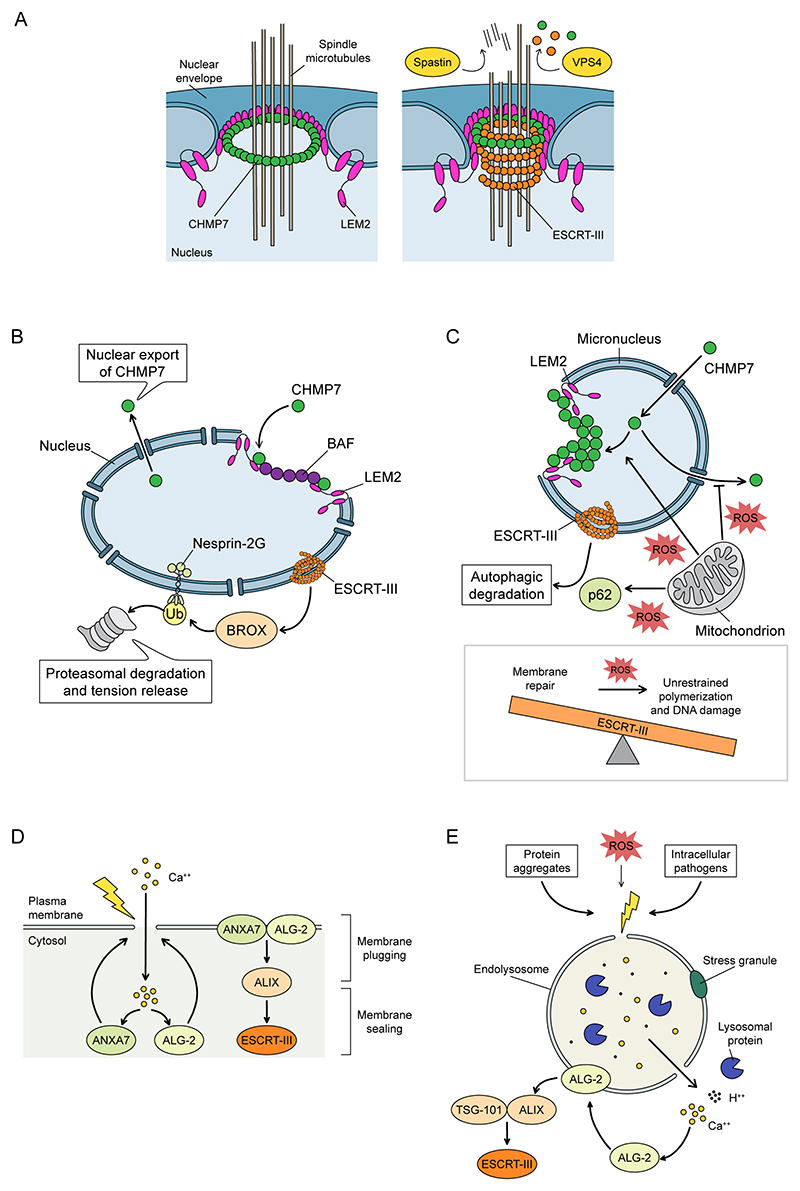
ESCRT activity during membrane repair. **A**. During exit from cell division, the inner nuclear membrane protein LEM2 recruits and activates charged multivesicular body protein 7 (CHMP7) to initiate ESCRT-III assembly and closure of holes in the reforming nuclear envelope. Through condensation on spindle microtubules that traverse the reforming nuclear envelope, LEM2 acts as a molecular O-ring to aid nuclear envelope sealing before these spindle microtubules are disassembled by the ESCRT-III-associated AAA-ATPase, Spastin and ESCRT-III filaments are remodelled by Vacuolar Protein Sorting-4 (VPS4). **B**. Damage to the nucleus can also occur during interphase. Such ruptures result in exposure of the DNA-binding protein barrier-to-autointegration factor (BAF), which coats the chromatin surface to restrict diffusion and can mobilise LEM2 and recruit CHMP7 to repair discontinuities in this membrane. The BroI-domain containing ESCRT-III-associated protein BROX is recruited to sites of interphase nuclear rupture where it facilitates the ubiquitination and degradation of Nesprin-2G, a nuclear envelope-associated protein that links to the cytoskeleton, releasing tension on the nuclear envelope and enabling membrane repair. **C**. Missegregated chromosomes that are not incorporated into the main nucleus during mitotic exit are enveloped by peripheral ER to become micronuclei. Micronuclear envelopes lack the normal complement of nuclear pore complexes and lamin proteins making them more fragile and prone to rupture. In addition to their role in repairing damage to these organelles, inappropriate CHMP7 import into micronuclei can lead to uncontrolled ESCRT-III polymerisation and micronuclear damage. Micronuclear accumulation, LEM2 binding and oligomerisation of CHMP7 can be enhanced by oxidation of cysteine residues in CHMP7 by reactive oxygen species (ROS) released from proximal mitochondria, limiting the ability of this machinery to repair micronuclei. ROS could also elevate p62-mediated autophagy of ESCRT-III components, which again limits the ability of this machinery to contribute to membrane repair. **D**. During plasma membrane repair, calcium influx mobilises the calcium and phospholipid binding Annexin proteins, including Annexin A7 (ANXA7) and its binding partner apoptosis linked gene-2 (ALG-2), which, through interaction with the ESCRT-III adaptor protein ALG-2-interacting protein X (ALIX), recruit downstream ESCRT-III proteins to the site of damage to orchestrate plasma membrane repair. **E**. Upon damage to lysosomes, ESCRT proteins and the adaptors ALG-2, mobilised by calcium release, are recruited to help repair the membrane, restore lysosomal integrity and limit activation of lysophagy pathways. Alongside ESCRT-dependent repair, a variety of other factors including membrane lipid metabolism and delivery, holes plugged by stress granules and ATG8-ylation of lysosomal membranes facilitate repair. These additional mechanisms of lysosomal repair are reviewed in^150^.

During late anaphase, mitotic chromosomes are progressively coated by the DNA-binding protein barrier-to-autointegration factor (BAF), proteins of the nuclear lamina and LEM-domain proteins (named for their founding members LAP2, emerin and MAN1), kickstarting nuclear envelope reformation^113–115^. Specifically, the LEM domain-containing protein LEM2 becomes enriched in proximity to the spindle microtubules due to binding of its low-complexity domain to BAF, leading to a phase separation event^35^ that promotes its loading onto spindle microtubules, and enables recruitment of CHMP7 to the resealing membranes^27,35,37,116^. The interaction between the winged-helix domains of both proteins induces a conformational change in CHMP7, which adopts an open conformation leading to its polymerisation and recruiting downstream ESCRT-III components for the final fusion event. The fact that LEM2 is enriched around spindle microtubules near chromatin in vivo and that CHMP7 and LEM2 copolymerize into ring-shaped filaments around microtubules in vitro are at the basis of the so-called “O-ring” model, in which the two proteins form a temporary macromolecular seal between the holes in the reforming nuclear envelope and the microtubules present in these fenestrations ^35^ (Figure 3A).

The fission yeast *Saccharomyces japonicus* makes use of an intranuclear mitotic spindle to separate its genetic material. At the end of this process, Lem2 (the yeast orthologue of LEM2) not only localises to the spindle pole body (SPB), a structure that is extruded from the nucleus after mitosis, but it also condenses at tails opposite the SPB, where the spindle microtubules intersect with the nuclear membrane to seal these ruptures. Cmp7 (the yeast orthologue of CHMP7) and ESCRT-III also localise to these zones, suggesting that coordination of cytoskeleton and membrane remodelling activities to close holes around disassembling microtubules are evolutionary conserved^117^. In *S. pombe*, which undergoes a closed mitosis, the extrusion of the SPB necessitates a membrane sealing event to maintain nuclear compartmentalisation. Here, a biomolecular condensate acts to limit nucleoplasmic loss, with ESCRT-III-dependent membrane closure occurring later in G1 (ref.^91^). Intriguingly, work in *S. pombe* has exposed a supplementary role for ESCRT-III during nuclear envelope reformation. In addition to its canonical sealing activity, this complex also restricts the size of the nuclear envelope holes formed by SPB extrusion^91^. In fact, depletion of Cmp7 results in bigger fenestrations (around 300 nm in diameter compared to less than 100 nm in the control condition), indicating that ESCRTs might also have a stabilising role to restrict the size of the holes and facilitate resealing.

#### Nuclear envelope repair during interphase

The repair of nuclear envelope ruptures during interphase seems to follow a mechanism similar to the postmitotic reassembly of the nuclear envelope^105,106^. Upon damage, a non-phosphorylated cytoplasmic pool of BAF rapidly accumulates near the exposed DNA. As the amount of BAF recruitment positively correlates with the severity of the nuclear envelope lesion^105,112^, this early event is thought to act as an initial plugging mechanism. BAF enrichment around the damaged area is then followed by re-localisation of LEM domain proteins (including LEM2), diffusion of CHMP7 into the nucleus and recruitment of downstream ESCRT proteins (Figure 3B). This process seems to proceed independently of the cause of the rupture^105^. However, it is not clear whether ESCRT-III activity is always required for resolving these lesions as BAF operating with LEM-domain proteins seems capable by itself to restrict diffusion across damaged nuclear membranes during interphase rupture^112^. At least in nuclear envelope reformation during cell division in *Caenorhabditis elegans*, it has been shown that only if BAF is unable to interact with LEM2, is the LEM2–CHMP7 dimer required to close holes around microtubules^118^, suggesting either that the BAF–LEM module is usually sufficient to plug these discontinuities, or that there are redundant mechanisms to perform nuclear envelope sealing. Interestingly, in contrast to the complete loss of nucleocytoplasmic compartmentalization reported in some models of nuclear envelope damage in mammalian cell lines^112^, depletion of BAF in *C. elegans* only results in a delayed repair and does not abolish it.

These conflicting results — whether BAF is required to recruit LEM-domain containing proteins and initiates ESCRT-III dependent repair, whether BAF is essential for completing repair, or whether ESCRT-III activity is necessary for sealing — strongly suggest that: 1) nuclear envelope sealing mechanisms are similar between nuclear envelope reformation and repair upon ruptures, although there could be some specific differences related to the cell cycle phase, the forces applied to the nucleus, or the nature of the hole to be sealed; 2) redundant mechanisms are in place and in specific contexts (depending on the nature of rupture, tissue, cycling or non-cycling cells, organism) some pathways are more important than others. For example, in yeast, Cmp7’s ability to interact with phosphatidic acid-rich membranes^119^ is necessary for recruitment to nuclear membranes. Although CHMP7’s membrane interaction is necessary for assembling it on mitotic nuclear membranes also in mammalian cells^116^, it is unclear whether a requirement for phosphatidic acid-rich membranes applies also for this system.

It has been recently reported that the ESCRT-III machinery has an additional role, specifically during the repair of ruptures of the interphase nucleus. Here, the ESCRT machinery can indirectly modulate the linker of nucleoskeleton and cytoskeleton complex (LINC), a system of nuclear envelope-associated proteins that transmits mechanical forces from the cytoplasm to the nucleus. Recruitment of the Bro1 domain-containing protein BROX by the ESCRT-III machinery to sites of nuclear envelope rupture targets the LINC component Nesprin-2G for ubiquitination and degradation, thus promoting relaxation of mechanical stress (Figure 3B). Since BROX depletion delays the reestablishment of nucleocytoplasmic compartmentalization upon nuclear envelope rupture, the ability of ESCRTs to remodel the LINC complex could be a mechanism to increase ESCRT-III’s membrane repair proficiency by minimising tension that counteracts the repair^120^.

It is thought that the LEM2–CHMP7 module can repair only small holes (<100 nm) in the nuclear envelope. Several observations lend indirect evidence to this hypothesis: CHMP2A preferentially decorates nuclear fenestrations of ~25–50 nm^110^, and Cmp7 hyperactivation leads to the formation of nuclear envelope herniations with a neck of ~45 nm diameter^121^. In addition, CHMP7 and LEM2 copolymerize in vitro, giving rise to spirals with a diameter of 50–100 nm^35^. These measurements are consistent with known diameters of the necks of budding HIV-1 virions, or intra-endosomal vesicle necks^122,123^ that ESCRT-III is known to sever. It may be that larger holes are stabilised by diffusion barriers with the progressive recovery of envelope until dimensions are suitable to mount an ESCRT-dependent seal.

#### Micronuclear envelope repair

Chromosomal instability, one of the hallmarks of cancer cells, is caused by DNA segregation errors and the subsequent formation of extranuclear DNA bodies called micronuclei^124^. Although enclosed by membrane, micronuclei contain aberrant nuclear envelopes with improper levels of NPCs and nuclear lamina compared to primary nuclei^125^. This leads to an inherent fragility, and damage to the micronuclear envelope is frequently irreversible, causing both chromothripsis and catastrophic chromosome rearrangements^125,126^. Analogously to that described for the membrane surrounding primary nuclei, ESCRT-III activity appears to be involved in resolving micronuclear lesions^127,128^. Yet, ESCRT-III activity is also one of the causes of micronuclear envelope fragility and DNA damage: CHMP7 is normally exported from the nucleus through C-terminal nuclear export sequences (NESs)^121,129^, however, at micronuclei, a defective export of CHMP7 leads to an unrestrained accumulation of ESCRT-III proteins, followed by micronuclear membrane deformation and, eventually, collapse^127,128^ (Figure 3C). Recent work has shed light on additional mechanisms underlying CHMP7 and ESCRT-III deregulation in these compartments^130,131^. Micronuclei have been found to make extensive contacts with mitochondria and this positively correlates with micronuclear collapse. The proximity between these organelles is thought to induce a local increase in reactive oxygen species (ROS) which interferes with ESCRT activity^130^. Mechanistically, high ROS levels result in defective export from the micronucleus, causing CHMP7’s micronuclear accumulation. Binding to LEM2 triggers unrestrained CHMP7 polymerisation which leads to micronuclear collapse, DNA damage and chromosome fragmentation. Moreover, ROS-induced oxidation of cysteines in CHMP7 promotes CHMP7 oligomerisation and generation of higher-order structures, which not only deform the micronuclear membrane but also hamper binding to other ESCRT-III components, contributing to micronuclear collapse. Additionally, the autophagy receptor p62 is recruited to micronuclear ruptures in a ROS-dependent manner and this results in the degradation of CHMP7, CHMP4B and CHMP2A, limiting the ability of the ESCRT machinery to repair these ruptured membranes^131^ (Figure 3C). Hence, CHMP7 and ESCRT-III activity has a dual role in micronuclear homeostasis and repair, promoting stability by sealing membranes as in other contexts, but inducing micronuclear envelope deformation when deregulated, thus abrogating micronuclear integrity and complicating the resolution of lesions.

#### Plasma membrane repair

Plasma membrane damage can arise from a wide range of sources, which can be categorized into mechanical insults, arising when physical forces are applied to the membrane, and biochemical insults, such as from pore-forming proteins and lipid peroxidation due to ROS. Several mechanisms of plasma membrane repair have been proposed and rely either on the removal of the damaged region or on the regeneration of the affected area through delivery of intracellular membranes. Membrane repair is initiated rapidly once a discontinuity forms in the lipid bilayer, triggered by a rapid influx of extracellular calcium ions^132^. Similarly to what happens during nuclear envelope sealing, the ESCRT-III machinery is thought to be engaged in the presence of small holes (< 100 nm)^133,134^, although ESCRT-III recruitment has been also described in the context of wider lesions (>1 μm)^134^. Upon generation of a plasma membrane wound, a calcium wave induces the recruitment of the calcium-binding proteins such as Annexin A7 (ANXA7) and apoptosis-linked gene 2 (ALG-2), which in turn recruits ALG-2-interacting protein X (ALIX). ALIX acts as an adaptor for the ESCRT-III proteins CHMP4B, CHMP2A, CHMP2B, CHMP3, CHMP1A (but not CHMP6) and VPS4A/B^133–135^, facilitating their recruitment and repair of the damaged membrane (Figure 3D). Also, the ESCRT-I component TSG101 is recruited^134^, although it is unclear what role upstream ESCRT proteins have in this process.

Circular arrays of ESCRT-III have been observed at the inner leaflet of the plasma membrane in unperturbed cells^42^, which alongside the observation that bacterial ESCRT-III-like Vipp1 rings “scan” monolayers for regions of damage^61^, and that ESCRT-III components accumulate at the plasma membrane in the absence of VPS4 activity^22^. Based on these findings it is tempting to speculate that a pool of ESCRT-III is engaged in a surveillance role to respond to discontinuities in this membrane as they arise.

Pore-forming proteins can disrupt cellular membranes by creating pores in lipid bilayers, eventually leading to cell death. These proteins are used by pathogens to attack host tissues (e.g., *α*-hemolysin of *Staphylococcus aureus* or listeriolysin O of *Listeria monocytogenes*) as well as by host cells to eliminate infected or abnormal cells (e.g., production of perforin, and gasdermin D by immune cells). ESCRT proteins are involved in suppressing the action of these damaging agents by repairing membranes^136,137^. The ability of ESCRT-III to dampen the activity of gasdermin during pyroptosis^137^ and to counteract perforin damage caused by cytotoxic T lymphocytes secretion^138^ underlie a new connection between ESCRT activity and the immune system, suggesting that these proteins could not only have an anti-inflammatory role but also be co-opted by tumour cells to restrict T-cell and natural killer cell action. Thus, a better dissection of how ESCRT activity is engaged and regulated upon biochemically-induced damage could have important implications in the understanding of immune evasion in cancer cells.

In addition, it has been recently reported that ESCRTs are able to contribute to plasma membrane repair through a novel pathway, which relies on the internalization of damaged membranes. In this process, the membrane protein lipopolysaccharide-induced tumor necrosis factor-alpha factor (LITAF) promotes internalisation and sequestration of compromised bilayer patches into MVBs independently of calcium influx^153^. Eventually, ESCRT-dependent MVB maturation leads to degradation of the pore-containing membranes or their secretion in the form of exosomes. This study highlights how ESCRT proteins can be engaged in membrane repair through different pathways that, in contrast to the classic plasma membrane repair, do not rely on calcium-sensing proteins, and involve internalization of the lesions and their processing by the endosomal compartment.

#### Endolysosomal membrane repair

Membranes of the endolysosomal compartment can be damaged by particulate matter (e.g., protein aggregates or crystals), ROS or intracellular pathogens (such as *Mycobacterium tuberculosis*). The maintenance of intact membranes in these organelles is crucial for the cell, as their rupture can cause calcium efflux, neutralization of lysosomal pH, leakage of hydrolases into the cytosol and intracellular diffusion of pathogens, causing not only severe dysfunction of endolysosomes themselves but also posing a threat to the integrity of other organelles. Endolysosomal membranes can also be damaged in a physiological context, or to allow for endocytic escape and cross presentation of internalised antigens in dendritic cells^139^. ESCRT-III proteins are recruited to damaged endolysosomes downstream of the calcium-binding protein ALG-2 and the ESCRT adaptors ALIX and TSG101 (refs. ^140–144^) and a reduction in tension associated with rupture may also facilitate this recruitment^145^. ESCRT-III recruitment to these damaged lysosomes is associated with their resealing and restoration of lysosomal function (Figure 3E). This activity helps limit the spread of pathogens and protein aggregates such as tau fibrils^146,147^ and makes them more resilient against osmotic stress^144^. However, the exact roles that ESCRT-III proteins have at endolysosomal rupture sites are still being elucidated and ESCRT-independent lysosomal repair pathways have been reported^148–150^. Different hypotheses describing their mechanism of action have been proposed, including inward budding of damaged areas and subsequent recycling, first-line plugging of lesions, and recruitment of other downstream repair enzymes^151^.

The ESCRT machinery has a role in restricting intracellular bacterial infections at two distinct levels. ESCRTs are initially engaged for the repair of endosome damage caused by the pathogenic action of these organisms during their cytosolic entry phase. If the bacteria cause extensive ruptures and can escape into the cytosol, a second-line defence mechanism is activated, in which a specialized type of autophagy (xenophagy) neutralises and eliminates the pathogens by degrading them through lysosomal activity. Xenophagolysosome integrity is maintained by ESCRT activity thanks to the recruitment of VPS4 downstream to a TOM1-like protein 2 (TOM1L2)–Ras-related protein Rab41 module^152^. As described below, ESCRT-III has established roles in autophagy, ensuring pathogen destruction.

### ESCRT-III function in autophagy

Autophagy is an essential cellular process that involves the controlled degradation and recycling of damaged or excess cytosolic components^154^. This can be achieved by engulfing material in a double membrane vesicle, which subsequently fuses with lysosomes (macroautophagy), by internalization of small portions of cytoplasm through invagination of the lysosome membrane itself (microautophagy) or by recognition and lysosomal translocation of specific protein substrates based on chaperones (chaperone-mediated autophagy). Macroautophagy is initiated by the formation of a double-membraned structure called the phagophore, which expands to engulf targeted cellular material until it fully encloses the cargo, giving rise to the autophagosome. Once sealed, this autophagosome then fuses with a lysosome, creating an autolysosome where the enclosed material is degraded by lysosomal enzymes and recycled.

**Figure 4:**
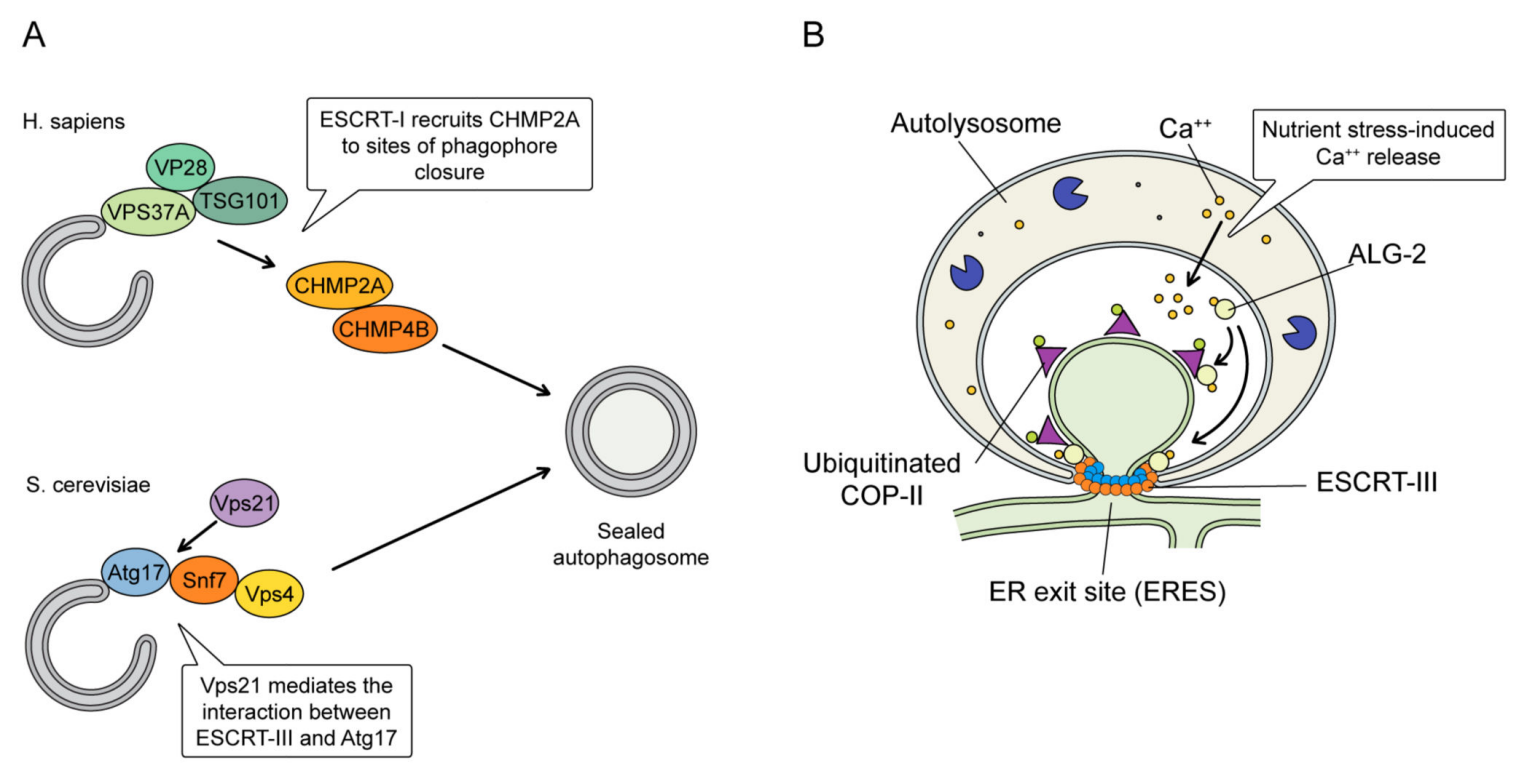
ESCRT-III roles in autophagy. **A**. During autophagosome closure, ESCRT components including the ESCRT-I subunits tumour susceptibility gene 101 (TSG101), vacuolar protein sorting-28 (VPS28) and VPS37A are recruited to the closing phagophore to enable the final sealing event. Autophagosome closure ensures that lumenal content is degraded upon fusion with the lysosome. In yeast, the GTPase Vps21 can also help promote interactions between ESCRT-III components and the autophagy receptor Atg17, facilitating ESCRT-dependent autophagy. **B**. ER exit sites (ERES) are major hubs through which secretory proteins are trafficked en-route to the Golgi apparatus. The ESCRT-associated protein ALG-2 can control coatomer-protein-2 (COP-II)-mediated ERES activity in a calcium-dependent manner. Nutrient stresses can lead to calcium triggered ALG-2 mobilisation at ERES, which allowed the engulfment of ERES in a ubiquitin- and ALIX-dependent manner, likely enabling the digestion and recycling of secretory proteins to relieve the nutrient stress.

The ESCRT-III machinery has a role in macroautophagy^155–157^ and disruption of CHMP2A or VPS4 activity results in the accumulation of phagophores and inhibition of their sealing^158,159^. A genome-wide CRISPR screen identified the ESCRT-I proteins VPS37A, TSG101 and VPS28 as components required for CHMP2A recruitment to sites of phagophore closure^160^, an event topologically equivalent to all other ESCRT-III-mediated membrane remodelling (Figure 1A). Consistent with this, CHMP4B localised transiently to the closing phagophore during mitophagy and was necessary for phagophore sealing (Figure 4A). In yeast, the Rab5 GTPase Vps21 is involved in localizing Snf7 and Vps4 to autophagosomes via the autophagy-related protein 1 (Atg1) complex component Atg17 (ref. ^161^) (Figure 4A). Without autophagosome closure, sequestered content would be released back to the cytosol upon fusion with the lysosome, demonstrating an essential function for ESCRT-III in autophagic proteostasis.

By combining live-cell super-resolution imaging, focused ion beam scanning electron microscopy (FIB-SEM) and an in vitro reconstitution assay, a recent study showed that ESCRT-III activity is also involved in microautophagy of endoplasmic reticulum exit sites (ERES), specialized regions of the ER that package nascent cargo proteins and lipids for the secretory pathway^162^. In conditions of nutrient stress (such as upon starvation or pharmacological inhibition of the mammalian target of rapamycin mTOR) calcium is released from lysosomes^163^, triggering the localization of the calcium-binding protein ALG-2 to ERES^164^. At these sites, ALG-2 binds membranes, the COP-II component, SEC31 and ALIX^143^. Here, ALG-2 is proposed to have a dual role: not only does it promote the association between ERES and autolysosomes, but via ALIX and ESCRT-III, it initiates invagination of ERES into the recruited lysosomes, thus facilitating their engulfment (Figure 4B). It is unclear how ERES are released from the bulk ER, but as this process was dependent upon ubiquitinated SEC31, this may involve coat protein II (COPII)-dependent budding. Lysosomal degradation of proteins moving through ERES is proposed to release amino acids for recycling under conditions of nutrient starvation.

### ESCRT-III function in cell division

**Figure 5:**
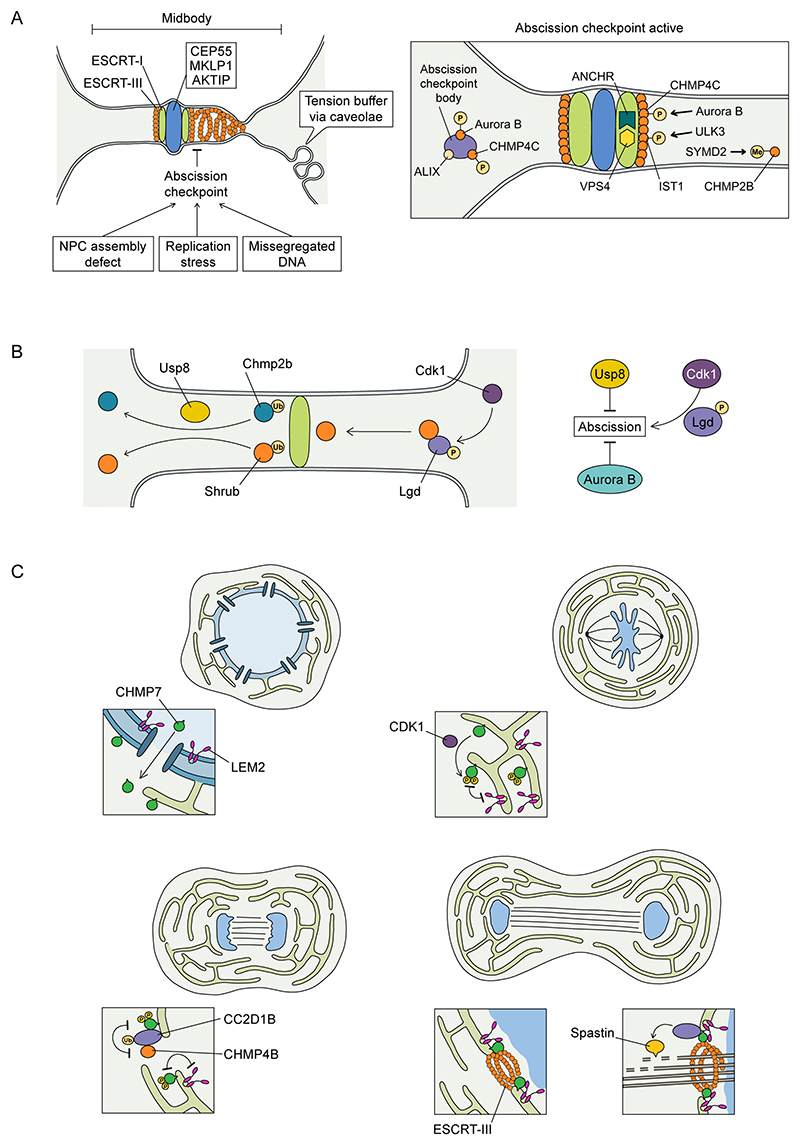
Regulation of ESCRT-III activity during cell division. **A**. The final stages of cell division require precise control of the activity of ESCRT-III. During cytokinesis, the centrosomal protein of 55 kDa (CEP55) and mitotic kinesin-like protein 1 (MKLP1) initially recruit ESCRT-III to the midbody of dividing cells via ALG-2-interacting protein X (ALIX) and tumour succeptability gene-101 (TSG101). The AKT-interacting protein, AKTIP can also bind VPS28 and assists ESCRT-III localisation to the midbody. Here, ESCRT-III severs the membrane connection through the process of abscission, allowing daughter cell individualisation and the completion of cell division. However, the activity of ESCRT-III at this site is regulated to ensure cytokinesis completes with appropriate timing. In mammalian cells, in response to a variety of cellular stresses, including replication stress, mis-segregated DNA, or nuclear pore complex (NPC) assembly defects, ESCRT-III-dependent abscission can be delayed through phosphorylation of the ESCRT-III component CHMP4C by Aurora B, preventing completion of abscission. Several other proteins contribute to this negative regulation of abscission, including the abscission/NoCut checkpoint regulator (ANCHR), and the ESCRT-III component IST-1 whose phosphorylation by unc 51-like kinase-3 (ULK3) and ability to recruit the protease Calpain-7 is necessary for abscission delay. Additionally, methylation of CHMP2B by the N-lysine methyltransferase SET and MYND Domain containing 2 (SMYD2) can relieve this checkpoint to accelerate abscission. **B**. During interphase, CHMP7 is actively exported from the nucleus to prevent its interaction with LEM2. During mitosis, cyclin-dependent kinase 1 (CDK1) phosphorylates CHMP7 to restrict its interaction with the inner nuclear membrane protein LEM2 and its ability to polymerise. These inhibitory phosphorylations are removed at the anaphase-to-telophase transition to license LEM2-stimulated CHMP7 polymerisation and ESCRT-III assembly at the reforming nuclear envelope. Additionally, CHMP7 recruits the CHMP4-binding protein Coiled-coil and C2 domain-containing protein 1B (CC2D1B) to the reforming nuclear envelope and is necessary for the timely recruitment of ESCRT-III to reforming nuclear membranes. Through CC2D1B and ESCRT-III, Spastin helps sever spindle microtubules. **C**. During cytokinesis in the *D. melanogaster* germline, ESCRT-III activity is needed for completion of abscission, and this activity is positively regulated by phosphorylation of the Shrub regulator, lethal giant discs (Lgd) by the mitotic kinase cyclin dependent kinase-1 (Cdk1). Ubiquitination was found to be necessary to localise Shrub to the abscission site and this process is physiologically regulated to allow cycles of incomplete division through the deubiquitination of Shrub and Chmp2B by the ESCRT-associated deubiquitinase ubiquitin-specific peptidase-8 (Usp8). This allows the formation of interconnected, multinucleate germline cysts.

ESCRTs have essential roles in cell division, specifically during the physical separation of daughter cells (cytokinesis) (Figure 5) and in the regeneration of the nuclear envelope, as described above (Figure 3A). Following the metaphase-to-anaphase transition, when the two chromosome masses are segregated, the equatorial region of the mitotic spindle is progressively constricted, giving rise to the midbody, which marks the future cleavage site to separate daughter cells. In this region, the centralspindlin complex^165^ and the mitotic kinesin-like protein 1 (MKLP1)-associated centrosomal protein of 55 kDa (CEP55)^166,167^ act as adaptors for the recruitment of the ESCRT machinery, which, through ESCRT-III and VPS4, mediates the membrane constriction and abscission events at the midbody. Whereas CEP55 is dispensable for many somatic divisions^168^, these additional adaptors likely provide alternate pathways of ESCRT-III recruitment to ensure completion of cell division. Although a central role of the ESCRT-binding protein ALIX has been demonstrated for the localization of ESCRT-III components, other studies have highlighted the presence of independent and/or redundant pathways to ensure coordination of ESCRT-III at this site^169,170^. ESCRT-III components assemble in large-diameter rings proximal to the midbody in early cytokinesis and later relocate to the site of abscission along the midbody arms^52,171^, an area characterised by cortical actin clearance^172,173^ and a secondary ingression that brings midbody membranes into close apposition. Midbody membranes are tethered to the narrowing ESCRT-III spiral via ALIX’s interaction with syndecan and syntenin^174^. Finally, abscission is integrated with microtubule severing through the activity of the ESCRT-III-associated AAA ATPase, Spastin, and actin-associated microtubule destabilisation at the abscission site^175–177^.

Whilst centralspindlin and ALIX recruit ESCRT-III during cytokinesis in *D. melanogaster* germline stem cells^165,178,179^, a recent study found that during oogenesis, lethal giant disc (Lgd), the homologue of coiled-coil and C2 domain-containing protein 1A/1B (CC2D1A/CC2D1B), also helps recruit Shrub, the CHMP4B homologue of *D. melanogaster*, to perform abscission^180^, suggesting alternate mechanisms for efficient recruitment of ESCRT-III and completion of cytokinesis.

ESCRT activity is central not only for the abscission step of cytokinesis but also for its regulation. The abscission checkpoint, a signalling module composed of the chromosomal passenger complex (CPC) component Aurora B and CHMP4C, has been shown to delay cytokinesis when lagging chromosomes are sensed in the intercellular bridge, preventing a catastrophic premature separation and thus safeguarding against aneuploidy^181,182^ (Figure 5A). This checkpoint is also engaged if NPC assembly is compromised^183^ or in the presence of replication stress^184^. The delay is mediated by sequestering ESCRT components in cytoplasmic granules called abscission checkpoint bodies^185^ and the retention of ESCRT-III proteins in central regions of the midbody away from the site of abscission^181,186^ (Figure 5A). ESCRT-III assembly in this region is also regulated by physical forces, with tension release promoting ESCRT-III polymerization at the midbody^187^. Caveolae, flask-shaped invaginations that act as a membrane store, have been shown to form at the midbody and abscission site where they buffer membrane tension and promote ESCRT-III recruitment and abscission^188^ (Figure 5A). Drawing analogies to BROX-mediated Nesprin-2G degradation that enables ESCRT-III-dependent nuclear envelope repair^120^, it is possible that membrane tension itself can restrict ESCRT-III-dependent membrane fission.

### Regulation of ESCRT-III activity

ESCRT-III activity needs to be controlled both in space and time, in order to avoid uncontrolled activation. In the following subsections we describe different regulatory mechanisms.

#### Control of conformation and localisation

The main level of regulation relies on the conformational status of the ESCRT proteins (closed vs open), which affects their ability to polymerise. For instance, it has been shown that the scaffold proteins CC2D1A, CC2D1B and the *D. melanogaster* homologue, Lgd, directly bind to the N-terminus of CHMP4B (Shrub in *D. melanogaster*)^189^ and inhibit CHMP4B polymerization in vitro^190,191^. As described below, this activity is deployed to ensure the timely assembly of ESCRT-III at sites of activity, perhaps by ensuring the delivery of polymerisation-restricted monomers that can be polymerised in a timely manner. A second level of regulation is achieved by priming ESCRT activity in a specific subcellular region thanks to the interaction with location-specific factors. For instance, viral Gag proteins help assemble ESCRT-III at sites of viral budding^123^ and, in the context of endosomal sorting, ESCRT-III polymerization is activated by interaction with upstream ESCRT components that are themselves localised to endosomes through the PtdIns(3)P-binding FYVE domain in HRS and Vps27 (refs. ^192,193^). Similarly, the ESCRT-III machinery is activated during cytokinesis thanks to the interaction between midbody-enriched proteins (CEP55, MKLP1, AKTIP), the ESCRT-I protein TSG101 and ALIX^166,167,194^ (Figure 5A). During nuclear envelope sealing, ESCRT activity is regulated through several mechanisms: CHMP7 recruitment at the reforming nucleus is mediated by the INM protein LEM2 and this interaction initiates ESCRT-III polymerization^37^. Moreover, CHMP7 recruits CC2D1B, which acts as a regulatory factor by coordinating ER deposition with timely CHMP4B recruitment and spastin-mediated microtubule severing^191^ (Figure 5B).

#### Control by post-translational modification

ESCRT-III activity is also modulated by post-translational modifications. Regulation of ESCRT activity by phosphorylation has been well studied in at least three events during mitotic exit. Firstly, in the context of nuclear envelope reformation in late anaphase, the upstream ESCRT-III component CHMP7 acquires affinity for LEM2 when two inhibitory cyclin-dependent kinase 1 (CDK1)-dependent phosphorylations are removed^129^ (Figure 5B). This helps maintain inactivity of the CHMP7–LEM2 module during early mitosis when both partners are present in the mitotic ER, and licenses the LEM2-dependent ESCRT-III assembly during mitotic exit when these inhibitory phosphorylations are removed. Secondly, Aurora B kinase phosphorylates CHMP4C at its C-terminus, activating the abscission checkpoint, which delays cytokinesis in the presence of mitotic errors^181,182^ (Figure 5A). Current models suggest that this post-translational modification could promote the re-localisation of CHMP4C to the central region of the midbody, where it prevents the recruitment and polymerization of other ESCRT-III proteins. Notably, CHMP4C’s phosphorylation site (Ser210) lies within a sequence not shared by CHMP4A or CHMP4B. This suggests a functional antagonism among isoforms, in which the membrane scission activity promoted by CHMP4B is restrained by phosphorylated CHMP4C until the abscission checkpoint conditions are fulfilled. Lastly, in a parallel arm of the abscission checkpoint, the unc-51-like kinase 3 (ULK3) kinase can phosphorylate IST1 to delay abscission, ensuring faithful cytokinesis progression when checkpoint conditions are satisfied^195^ (Figure 5A). Additional contributions to the abscission checkpoint are made by the abscission/NoCut checkpoint regulator (ANCHR)^186^ and the protease Calpain-7, which localises to midbodies downstream of IST-1 and whose protease activity is necessary for abscission delay through this checkpoint^196^. The abscission checkpoint appears to operate by restricting ESCRT-III proteins localising to the abscission site, retaining them either at the midbody ring or in abscission checkpoint bodies (Figure 5A).

Although phosphorylation of ESCRT components seems to restrict their activity, Cdk1-phosphorylation of Lgd can enhance abscission in germline stem cells^180^. Given Lgd’s role in restricting Shrub polymerisation, this phosphorylation may relieve this activity (Figure 5C). Indeed, in the context of nuclear envelope reformation, ESCRT-III assembly is advanced in cells lacking CC2D1B, suggesting that these proteins control timely assembly of ESCRT-III at several stages during division^191^. Finally, a recent study has revealed that ESCRT-III activity during cytokinesis is subjected to an additional layer of regulation, where the timely CHMP2B relocation to the midbody and abscission was dependent on its methylation by the lysine methyltransferase SET and MYND domain-containing protein 2 SMYD2 (ref ^197^) (Figure 5A). Methylation of CHMP2B enhanced ESCRT-dependent abscission and countered the abscission checkpoint, raising the exciting possibility that different classes of post-translational modifications are coordinated to control the activity of this complex.

During receptor endocytosis, CHMP1B was found to be transiently ubiquitinated after epidermal growth factor (EGF) stimulation^198^, suggesting that this post-translational modification occurs concomitantly with ESCRT-III activation on endosomes. The deubiquitinase ubiquitin-specific-processing protease 8 (USP8) removed these ubiquitin moieties suggesting that dynamic addition and removal of ubiquitin could also be a way to modulate ESCRT-III activity. Indeed, in *D. melanogaster*, the ESCRT-III proteins Chmp2B and Shrub were found to be ubiquitinated and this mediated their midbody targeting during cytokinesis in germline stem cells. These proteins could be deubiquitinated by Usp8, and this prevented midbody localisation and promoted the incomplete divisions in stem cells that are necessary for formation of stem-cysts in the *D. melanogster* germline^199^ (Figure 5C).

Finally, selective proteolysis has also been shown to regulate ESCRT-III activity. In the crenarchaeon *Sulfolobus acidocaldarius*, division rings formed by the ESCRT-III proteins cell division protein B (CdvB), CdvB1 and/or CdvB2 (together referred to as CdvB1/CdvB2) enable cytokinesis to proceed^200,201^. Here, proteolysis of CdvB allows constriction of the CdvB1/CdvB2 division ring to facilitate abscission^202^, highlighting regulated proteolysis as an additional way of controlling subunit exchange and driving ESCRT-III to completion. As proteolysis is used extensively during the cell cycle to ensure unidirectionality, it may be that this form of regulation of ESCRT-III is a simple way to achieve switch-like behaviour when triggering the final division event. In mammalian cells, the cysteine protease Calpain-7 is recruited to midbodies by IST1 and its protease activity is necessary for ESCRT-III-dependent cytokinesis^196^. Although its substrates are unknown, these data indicated that targeted degradation or proteolytic processing may have wider roles in ESCRT-III remodelling and ESCRT-dependent membrane fission.

## Conclusions and perspectives

ESCRT-III is an ancient membrane remodeling complex. Its presence in the last universal common ancestor and its retention throughout evolution highlight its importance to cells. This importance likely stems from the topological uniqueness of the membrane-severing event that it performs: whilst there are many cellular machineries — such as Bin-Amphiphysin-Rvs (BAR)-domain containing proteins, coatomer proteins and dynamins — that could promote membrane deformation and severing of buds projecting into the cytoplasm, ESCRT-III appears unique in its ability to perform the reverse reaction, severing membranes projecting out of the cytoplasm and separating membranes that were previously connected (Figure 1). It is striking how this complex has been deployed throughout evolution as cellular complexity increased. We are now refining our understanding of the molecular events underlying ESCRT-III-dependent membrane remodeling and resultant membrane fission. Our next steps ought to determine how holo-filaments of ESCRT-III assemble and are remodeled in cells, and how this complex is regulated and functions alongside other cellular machineries to ensure that membrane remodeling occurs at precisely the right place and right time. Given the diverse range of cellular membranes that ESCRT-III can act on, one area that has received relatively little attention is our understanding of the lipid environment that permits ESCRT-III-dependent membrane remodeling. Although individual ESCRT-III monomers bind negatively-charged membrane lipids, and alterations in the presentation of membrane binding surfaces in ESCRT-III polymers are instrumental in allowing filaments to adapt to, and even drive, alterations in membrane geometry, it is not clear whether any specific lipids are needed to allow ESCRT-III-dependent membrane remodeling to occur. The next few years promise to illuminate how this fascinating protein complex polymerises to shape and separate membranes, and how cells employ this activity to control a wide range of their physiology.

## Supplementary Material

Supplementary Table 1

## Figures and Tables

**Table 1:** List of ESCRT subunits and associated proteins in mammals, yeast and flys. Protein names for ESCRT subunits and their aliases were recovered from Uniprot and Flybase. Uncharacterised genes in *D. melanogaster* that are suggested orthologues are written in brackets. Abbreviations are listed: ALG-2 interacting protein X (ALIX); apoptosis linked gene-2 (ALG-2); associated molecule with the SH3 domain of STAM (AMSH); Bck-like resistance to osmotic shock (Bro); BroI-domain containing protein-X (BROX); breast cancer-2 (BC2); charged multivesicular body protein (CHMP, CHM, CMP); coiled coil and C2-domain containing (CC2D); degradation of alpha (Doa); Doa4-independent degradation (Did); ELL associated protein (EAP); endosomal sorting complex required for transport (ESCRT); Hbp, STAM and EAST (Hse); hepatocyte growth factor regulated tyrosine kinase substrate (HRS); his-domain containing tyrosine phosphatase (HD-PTP); increased sodium tolerance (IST); lethal giant discs (Lgd); lysosomal-trafficking regulator interacting protein (LIP); microtubule interacting and trafficking domain-1 (MITD1); Mos10: more of Ste6; multivesicular body subunit (MVB); programmed cell death (PDCD); programmed cell death-6 interacting protein (PDCD6IP); protein tyrosine phosphatase, non-receptor type (PTPN); signal transducing adaptor molecule (STAM); STAM binding protein (STAMP); sucrose non-fermenting (Snf); suppressor of K+ transport growth defect (SKD1); tumour susceptibility gene-101 (TSG101); ubiquitin associated protein (UBAP); ubiquitin-specific peptidase (USP, UBP); vacuolar protein sorting (VPS); vacuolar protein sorting-associated protein (VTA).

	Mammals	Mammals Aliases	Yeast	Flys
	HRS		Vps27, Did7	Hrs
ESCRT-0	STAM1		Hse1	Stam
	STAM2			
	TSG101		Vps23	Tsg101
	VPS28		Vps28	Vps28
	VPS37A		Vps37	Vps37A
	VPS37B			Vps37B
ESCRT-I	VPS37C			
	VPS37D			
	MVB12A		Mvb12	Mvb12
	MVB12B			
	UBAP1			
	EAP20	VPS25	Vps25	Vps25
ESCRT-II	EAP30	VPS22	Vps22, Snf8	Vps22, Larsen
	EAP45	VPS36	Vps36	Vps36
	CHMP1A		Vps46, Did2, Chm1	Chmp1
	CHMP1B			
	CHMP2A	BC2	Vps2, Did4, Chm2	Vps2
	CHMP2B			
	CHMP3		Vps24, Did3	Vps24
	CHMP4A		Vps32, Did1, Snf7,	Shrub
ESCRT-III	CHMP4B			
	CHMP4C			
	CHMP5		Vps60, Chm5, Mos10	Vps60
	CHMP6		Vps20, Chm6	Vps20
	CHMP7		Chm7, Cmp7	(CG5498)
	IST1		Ist1	Ist1
	VPS4A		Vps4, Did6	Vps4
VPS4 complex	VPS4B	SKD1		
	VTA1	LIP5	Vta1	
	USP8	UBPY	Doa4	
Deubiquitinases	STAMBP	AMSH		
	ALIX	PDCD6IP	Vps31, Bro1	ALiX
	HD-PTP	PTPN23		
ESCRT- associated	ALG-2	PDCD6		
	MITD1			(CG30398)
	CC2D1A			Lgd
	CC2D1B			
	Spastin			Spastin

## Box 1 ESCRT-related pathologies

As ESCRT-III proteins play crucial roles in various cellular processes, their loss of function or mutation has been linked to numerous pathologies, including neurodegenerative disorders, viral infections, and cancer.

### Neurodegenerative Disorders

Dysregulation of ESCRT-III proteins has been implicated in the pathogenesis of several neurodegenerative disorders. In particular, mutations in *CHMP2B* are linked to the onset of frontotemporal dementia (FTD)–amyotrophic lateral sclerosis (ALS) spectrum disorders. Mutant forms of CHMP2B have been shown to disrupt the correct functioning of the endolysosomal and autophagic degradation system, leading to the accumulation of misfolded proteins, inducing cellular stress and eventually resulting in neuron death^203,204^. Interestingly, CHMP2B-driven neurodegeneration lacks stereotypical neuropathological marks, such as TAR DNA-binding protein 43 (TDP-43), fused in sarcoma (FUS) or tubulin associated unit (TAU) inclusions^205^. However, CHMP2B can modulate TDP-43 phosphorylation by influencing the protein levels of casein kinase 1 (CK1)^206^, suggesting the presence of incompletely understood interactions between CHMP2B, TDP-43 and FTD–ALS pathogenesis. CHMP2B is also involved in the maintenance of synaptic morphology and functionality^207,208^, suggesting that the toxicity associated with C-terminally truncated mutants of this protein could be due to defects in both vesicle trafficking and membrane homeostasis^209^. CHMP7 has also been linked to ALS, as it accumulates in the nuclei of ALS-affected neurons. This in turn induces disruption of nuclear pore complex (NPC) integrity, reduction of nucleoporin expression, TDP-43 mislocalization and, eventually, neuronal degeneration^210^. CHMP7 nuclear influx is an early pathogenic event influencing the two main ALS pathological features (NPC injury and TDP-43 disfunction), suggesting a potential therapeutic strategy for ALS. Additional neurodevelopmental disorders related to ESCRT dysfunction include a multisystem neurodevelopmental disease called CIMDAG (for cerebellar hypoplasia and cataracts, intellectual disability, congenital microcephaly, dystonia and dyserythropoeitic anemia, growth retardation) caused by de novo mutations in *VPS4A*^211^. It remains to be fully understood why deregulation of ESCRT components involved in non-tissue-specific functions predominantly affects the nervous tissue. It could be hypothesized that, as neurons are highly polarized cells with an extensive plasma membrane, and because ESCRT-III proteins are also involved in membrane repair, this could make neurons particularly vulnerable to damage and failure of ESCRT proteins to efficiently maintain membrane homeostasis could exacerbate neurodegeneration by allowing sustained damage and loss of cellular integrity.

### Viral Infections

Several enveloped viruses (including HIV-1 and Hepatitis B) can hijack the ESCRT pathway to facilitate viral spreading through budding and release from host cells^212^. In HIV-1 biogenesis, the Gag protein interacts with many components of the ESCRT machinery, recruiting TSG101 and ALIX to the site of viral budding and then the ESCRT-III proteins CHMP2, CHMP3 and CHMP4. This leads to the formation of stereotypical ESCRT filaments which, in coordination with the action of the Gag lattice, are thought to facilitate viral budding and release (Figure 1A). Even if Gag has been shown to establish direct binding with several upstream ESCRT proteins, for ESCRT-III, HIV-1 release is strictly dependent on the activity of CHMP2 and CHMP4 proteins only^95^. Thus, a targeted blocking of the interaction between these ESCRT components and viral proteins has been proposed as a potential therapeutic strategy to reduce viral spread. Remarkably, truncated retrocopies of CHMP3 have been identified in New World monkeys and mice that appear to poison ESCRT-III-dependent viral budding while sparing cellular ESCRT-functions such as cytokinesis^213^. ESCRT-III is also recruited to the inner nuclear membrane (INM) to allow budding of progeny Herpes-Simplex-1 (HSV-1) virions into the intermembrane space for subsequent fusion with the outer nuclear membrane and release into the cytoplasm^214^ (Figure 1A).

### Cancer

Due to the involvement of ESCRT proteins in many different cellular processes, the deregulation of this machinery has the potential to promote tumour proliferation impairing receptor trafficking, disrupting cell signalling, inducing escape from apoptosis or supporting abnormal cell division. However, even if alteration of ESCRT levels has been reported for several tumour types in different model organisms, in most cases these do not directly translate into an increase in tumorigenic potential (reviewed in ref. ^215^) and it is possible that ESCRT loss is incompatible with cellular survival. Given the essentiality of ESCRT proteins, it is noteworthy that inactivation of *VPS4B* due to a loss of heterozygosity on chromosome 18 is observed frequently in colorectal cancer, rendering cells exquisitely sensitive to VPS4A inhibition^216,217^. This finding might lead to targeted therapies for this cancer that exploit the synthetic lethality between these proteins. Colorectal cancers also display frequent loss of VPS37 subunits, destabilising ESCRT-I and leading to impaired downregulation of signalling receptors and NF-kB-driven inflammation^218^. In addition, even though ESCRT proteins exert their activity as multiprotein complexes, deregulation of different subunits has been reported to result in opposing effects, whereby some components seem to act as tumour suppressors and others as oncoproteins^215,219–223^. For CHMP4C, a polymorphism associated with enhanced susceptibility to ovarian cancer impairs the function of the abscission checkpoint, leading to the development of aneuploidy^224^. Moreover, the contribution of ESCRT proteins to tumorigenesis could be due to functions independent of classical ESCRT roles, as seen for CHMP5’s involvement in establishing and maintaining a malignant transcriptional programme in T-cell acute lymphoblastic leukaemia^225^. Through their ability to sort cargo onto intra-endosomal vesicles (IEVs) that can be released as exosomes, ESCRTs have the ability to modulate the secretome^226,227^, which can reprogramme the tumour microenvironment to facilitate or restrict oncogenesis. However, as the IEVs themselves are intermediates on the degradation pathway, it has been unclear how cells decide whether to degrade or secrete the contents of their MVBs. Recently, this axis was shown to be controlled by ALIX-dependent F-actin polymerisation that favoured trafficking of MVBs to the periphery to promote exosomal release^228^, highlighting one mechanism by which IEV fate might be determined.

### Cataracts

Mutations in the *CHMP4B* gene underlie rare, inherited forms of cataracts^229^, which are suggested to occur due to a failure in ESCRT-dependent cytokinesis^230^, the clearance of extranuclear chromatin^231^ and/or impaired lens growth and differentiation^232^. As such, these specialised tissues may be particularly sensitive to ESCRT impairment.

## Glossary

### Abbreviations

Coarse-grained simulations: Modelling techniques that simplify complex systems by grouping atoms or molecules into larger units (called beads), enabling the study of larger assemblies and longer timescales.
Laminopathies: A group of rare genetic disorders caused by mutations in genes encoding nuclear lamina protein, leading to abnormalities in nuclear shape and structure, and resulting in conditions such as progeria syndromes and muscular dystrophies.
Midbody: A transient, protein-rich structure that assembles at the intercellular bridge during cytokinesis in mammalian cells.
Open mitosis: A type of cell division in higher eukaryotes where the nuclear envelope completely breaks down to allow chromosome segregation.
Stochastic optical reconstruction microscopy (STORM): A super-resolution imaging technique that uses the random switching of fluorescent molecules on and off to reconstruct highly detailed images beyond the diffraction limit.
