# Supplementary material for: ESCRT-III function in membrane fission and repair
Source: Nat Rev Mol Cell Biol. Author manuscript; Available in PMC 2026 Apr 1. (PMC7618577; doi:10.1038/s41580-025-00909-1)
Supplement: Supplementary Table 1 [file EMS211648-supplement-Supplementary_Table_1.pdf]

nature reviews molecular cell biology
https://doi.org/10.1038/s41580-025-00909-1

Supplementary information

ESCRT-III function in membrane fission and repair

In the format provided by the authors and unedited

ESCRT-III function in membrane fission and repair

Burigotto, M.[1,2] & Carlton, J.G.[1,2]

1. Organelle Dynamics Laboratory, The Francis Crick Institute, 1 Midland Road, London, NW1 1AT
2. School of Cancer & Pharmaceutical Sciences, King's College London, SE1 1UL

Correspondence to: jeremy.carlton@kcl.ac.uk

Supplementary Table 1:

	Mammals	Mammals Aliases	Yeast	Flys
ESCRT-0	HRS		Vps27, Did7	Hrs
	STAM1<br>STAM2		Hse1	Stam
ESCRT-I	TSG101		Vps23	Tsg101
	VPS28		Vps28	Vps28
	VPS37A<br>VPS37B<br>VPS37C<br>VPS37D		Vps37	Vps37A<br>Vps37B
	MVB12A<br>MVB12B<br>UBAP1		Mvb12	Mvb12
ESCRT-II	EAP20	VPS25	Vps25	Vps25
	EAP30	VPS22	Vps22, Snf8	Vps22, Larsen
	EAP45	VPS36	Vps36	Vps36
ESCRT-III	CHMP1A<br>CHMP1B		Vps46, Did2, Chm1	Chmp1
	CHMP2A<br>CHMP2B	BC2	Vps2, Did4, Chm2	Vps2
	CHMP3		Vps24, Did3	Vps24
	CHMP4A<br>CHMP4B<br>CHMP4C		Vps32, Did1, Snf7,	Shrub
	CHMP5		Vps60, Chm5, Mos10	Vps60
	CHMP6		Vps20, Chm6	Vps20
	CHMP7		Chm7, Cmp7	(CG5498)
	IST1		Ist1	Ist1

VPS4 complex	VPS4A		Vps4, Did6	Vps4
	VPS4B	SKD1		
	VTA1	LIP5	Vta1	
Deubiquitinases	USP8	UBPY	Doa4	
	STAMBP	AMSH		
ESCRT-associated	ALIX	PDCD6IP	Vps31, Bro1	ALiX
	HD-PTP	PTPN23		
	ALG-2	PDCD6		
	MITD1			(CG30398)
	CC2D1A			Lgd
	CC2D1B			
	Spastin			Spastin

Table 1

List of ESCRT subunits and associated proteins in mammals, yeast and flys. Protein names for ESCRT subunits and their aliases were recovered from Uniprot and Flybase. Uncharacterised genes in *D. melanogaster* that are suggested orthologues are written in brackets. Abbreviations as follows: ALG-2 interacting protein X (ALIX); apoptosis linked gene-2 (ALG-2); associated molecule with the SH3 domain of STAM (AMSH); Bck-like resistance to osmotic shock (Bro); BroI-domain containing protein-X (BROX); breast cancer-2 (BC2); charged multivesicular body protein (CHMP, CHM, CMP); coiled coil and C2-domain containing (CC2D); degradation of alpha (Doa); Doa4-independent degradation (Did); ELL associated protein (EAP); endosomal sorting complex required for transport (ESCRT); Hbp, STAM and EAST (Hse); hepatocyte growth factor regulated tyrosine kinase substrate (HRS); his-domain containing tyrosine phosphatase (HD-PTP); increased sodium tolerance (IST); lethal giant discs (Lgd); lysosomal-trafficking regulator interacting protein (LIP); microtubule interacting and trafficking domain-1 (MITD1); Mos10: more of Ste6; multivesicular body subunit (MVB); programmed cell death (PDCD); programmed cell death-6 interacting protein (PDCD6IP); protein tyrosine phosphatase, non-receptor type (PTPN); signal transducing adaptor molecule (STAM); STAM binding protein (STAMP); sucrose non-fermenting (Snf); suppressor of K+ transport growth defect (SKD1); tumour susceptibility gene-101 (TSG101); ubiquitin associated protein (UBAP); ubiquitin-specific peptidase (USP, UBP); vacuolar protein sorting (VPS); vacuolar protein sorting-associated protein (VTA).
